# A hit for base editing: treatment of developmental epilepsy in a mouse model

**DOI:** 10.1172/JCI200689

**Published:** 2026-02-02

**Authors:** Sophie F. Hill, Ethan M. Goldberg

**Affiliations:** 1 Division of Neurology, Department of Pediatrics and; 2 The Epilepsy Neurogenetics Initiative, The Children’s Hospital of Philadelphia, Philadelphia, Pennsylvania, USA.; 3 Department of Neuroscience and; 4 Department of Neurology, The University of Pennsylvania Perelman School of Medicine, Philadelphia, Pennsylvania, USA.

## Abstract

CRISPR/Cas9 base editing holds the potential to treat disease caused by single-nucleotide variants. In contrast with conventional CRISPR/Cas9 approaches, base editing enzymatically induces precise DNA alterations and can directly correct disease-causing variants. In this issue of *JCI*, Reever et al. used base editing to treat a mouse model of a severe neurodevelopmental disorder caused by a pathogenic missense variant in the voltage-gated sodium channel gene *SCN8A*. This work represents a starting point for the further refinement of base editing to treat genetic epilepsy.

## Base editing: advanced genome editing technology

Naturally occurring Cas9 proteins are enzymes that induce double-stranded DNA or RNA breaks at sequences specified by a guide RNA. Double-stranded DNA breaks are usually repaired by nonhomologous end joining, an error-prone pathway that often induces random insertion/deletions (“indels”), which can disrupt the reading frame of the target gene. Most human applications of first-generation CRISPR technology deliberately introduce indels into the disease-causing gene (or a regulatory protein) to “turn off” the gene.

**Figure 1:**
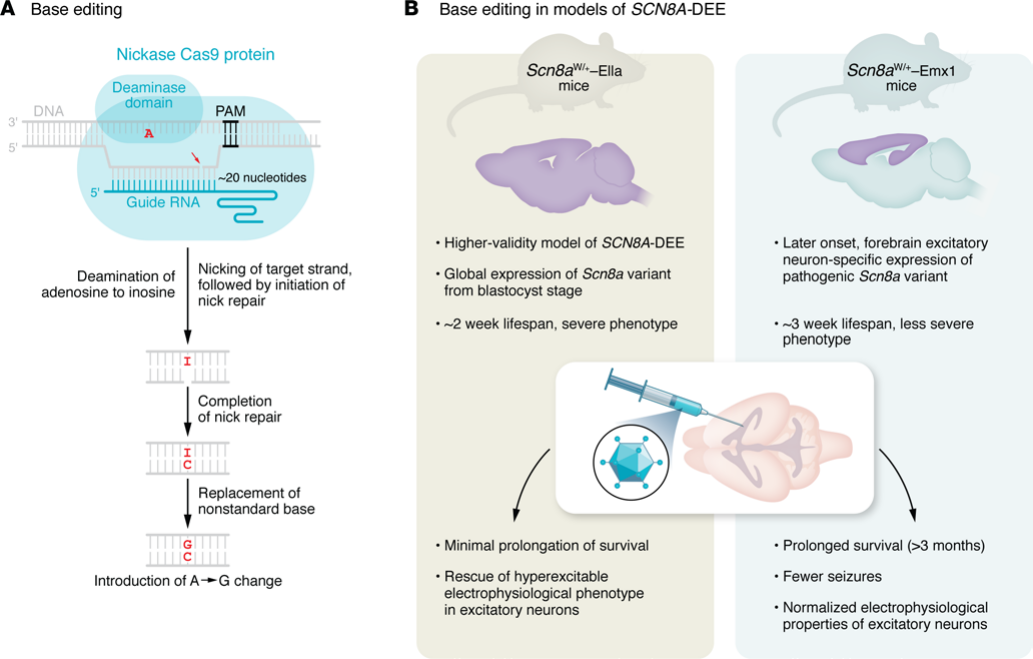
CRISPR/Cas9 base editing in a mouse model of SCN8A-related seizure disorder. (**A**) Base editing facilitates precise enzymatic correction of a mutant allele. The base editor is directed to a DNA locus by a guide RNA, and enzymatically changes nucleotide identity at that site. In this case, Reever et al. used adenine base editing to deaminate adenine into inosine, a nonstandard DNA base that is read as guanine. The mismatched I-T pair is detected and corrected to either the original A-T pair or the desired G-C pair. Nicking of the nonedited strand facilitates repair to G-C. (**B**) Reever et al. tested a base-editing strategy in two models of SCN8A-DEE by injecting the base editor packaged in an AAV vectors into the lateral ventricles on postnatal day 2 (16). In Scn8a^W/+^-EIIa mice, which express mutant Scn8a globally and display a severe phenotype and limited lifespan, the base editor treatment rescued the hyperexcitable phenotype in excitatory neurons but did not substantially extend lifespan. In Scn8a^W/+^-Emx1 mice, in which the mutant allele is restricted to forebrain excitatory neurons and has later-onset expression, base editor treatment prolonged survival, reduced seizure frequency, and normalized excitatory neuron function. Pam, protospacer adjacent motif.

Newer approaches use modified versions of Cas9 to perform more advanced genome editing without inducing double-stranded breaks. In particular, base editing was developed by the laboratory of David Liu to achieve enzymatic changes in nucleotide identity (Figure 1A) (1, 2). Base editors are fusion proteins composed of a catalytic domain and “nickase” Cas9. The catalytic deaminase domain converts either cytidine to uracil (read as thymine by polymerases) or adenine to inosine (read as guanine) on one strand. Cellular machinery detects the resulting mismatch and attempts to repair it, either by reverting to the original sequence or by replacing the unedited base to match its edited pair. The “nickase” Cas9 induces a single-stranded DNA “nick” on the unedited strand, promoting repair to match the edited base. The Liu lab has engineered improved base editors such that an ever-increasing proportion of single-nucleotide variants can be targeted and changed by a base editor (2, 3). These editors have proven effective in several preclinical models of human disease (4, 5).

In 2022, base editing was used in humans for the first time to treat inherited familial hypercholesterolemia by targeting *PCSK9*, which encodes a liver enzyme involved in lipoprotein homeostasis. The base editor reduced LDL cholesterol in all patients assessed, but two patients experienced serious cardiac events attributed to the underlying disease, reducing enthusiasm (6, 7). A recent successful trial in an infant with a life-limiting liver disease has raised hopes again (8, 9). Baby KJ Muldoon was diagnosed with CPS1 deficiency, a genetic disorder causing hyperammonemia, in the first month of life. Within 6 months, researchers at the Children’s Hospital of Philadelphia and University of Pennsylvania Perelman School of Medicine developed a personalized base editor to target his genetic variant, packaged it into lipid nanoparticles, and delivered it to the patient intravenously (8, 9). Following treatment, KJ’s physicians were able to reduce the dosage of his other medications and increase protein intake. Successful treatment on such a short time scale is an encouraging sign for the future of base editing therapeutics.

In both of the above examples, base editors were directed to the liver, which is readily targeted by intravenous administration. However, base editors have not yet been used to treat human neurological disease, in part due to the challenges of delivering large fusion proteins to the brain while avoiding liver toxicity. At the time of writing, no publication has reported a method to safely and efficiently cross the human blood-brain barrier, so therapies cannot be administered systemically and instead must be packaged into viruses and injected into the cerebrospinal fluid or directly into the brain parenchyma.

## Gene therapy in sodium channelopathies

Electrical signaling in neurons depends on the precise gating of ion channels. Genetic variants affecting ion channel proteins are common causes of neurological disease. Sodium channels, which mediate the upstroke of the action potential, are implicated in epilepsy and other disorders of electrical excitability. There are four brain-expressed sodium channel genes: *SCN1A* (encoding Nav1.1), *SCN2A* (encoding Nav1.2), *SCN3A* (encoding Nav1.3), and *SCN8A* (encoding Nav1.6). Sodium channel variants are generally classified as either “loss of function” or “gain of function” (GoF), depending on the effects on sodium current. GoF variants in *SCN8A* cause *SCN8A-*related developmental and epileptic encephalopathy (*SCN8A*-DEE), a severe seizure disorder defined by treatment-resistant epilepsy, movement disorder, and severe developmental delay/intellectual disability.

There have been several attempts to treat mouse models of *SCN8A-*related encephalopathy in mice with *Scn8a* GoF variants. In the *Scn8a^N1768D/+^* mouse, the small-molecule sodium channel blockers GS-967 and NBI-921352 reduced electrophysiological abnormalities in neurons, protected against electrically induced seizures, and extended survival (10, 11). Antisense oligonucleotides (ASOs), short DNA or RNA oligomers that can alter gene expression, have also extended the lifespan of *Scn8a* GoF mice while preventing seizures (12).

Similar approaches have been effective in mice with mutations in other sodium channel genes. The *SCN1A*-targeting ASO (which removes a “poison exon” to increase Nav1.1 expression from the intact allele) completed a Phase 1/2a clinical trial with promising results (13). CRISPR activation, wherein Cas9 is fused to a transcriptional activator to increase expression of target genes, has also improved the phenotypes of *Scn1a* and *Scn2a* mutant mice (14, 15). In this issue of *JCI*, Reever et al. described the first application of base editing to a mouse model of a genetic epilepsy (16).

## Base editing to treat *SCN8A*-DEE

Reever et al. assessed the efficacy and specificity of a series of guide RNAs and base editors in cultured cells to identify lead candidates to correct the mutant *Scn8a* allele (16). The best performing guide RNA and base editor combination, which was used for subsequent in vivo studies, exhibited a modest, approximately 30%, correction of the mutant allele; base editors to treat other disorders in mouse have achieved over 80% efficiency in cultured cells (4, 17).

Base editing machinery is too large to fit into standard AAV vectors. To deliver the base editor in vivo, the authors used the now-standard approach of splitting the editor into two viruses (4, 5). With a “split-intein” method, cells that take up both viruses can assemble the components of base editing machinery into a single protein. Reever et al. administered these viruses via injection into the lateral ventricles on postnatal day 2, achieving brain-wide expression of a GFP reporter. They administered the base editor to the Cre-dependent *Scn8a*^flox(R1872W)/+^ (W/+) mouse. Experimental mice were generated by crossing to a global Cre driver expressed from the blastocyst stage (*Scn8a*^W/+^-EIIa) or a forebrain excitatory neuron-specific Cre with later-onset expression (*Scn8a*^W/+^-EMX1) (Figure 1B).

Sham-treated *EIIa-*W/+ mice had a profoundly shortened lifespan, around two weeks. The base editor did not prolong survival in these mice, and the authors implied that there was insufficient time for therapeutic expression of the AAV/base editing machinery before death (16). However, the base editor did rescue the hyperexcitable electrophysiological phenotype associated with *Scn8a*^W/+^-EIIa excitatory neurons. Therefore, an alternative explanation could be that correction was not achieved in enough cells and/or brain areas. Supporting this view, the authors noted that mice with a higher proportion of edited cells survived longer. Absence of a survival benefit in these mice is somewhat concerning, since the *Scn8a*^W/+^-EIIa global mouse mutant has the highest validity as a model of *SCN8A-*related encephalopathy in human patients, who harbor *SCN8A* variants in all cells. In human and mouse, Nav1.6 is expressed in both excitatory and inhibitory (GABAergic) neurons and in brain areas beyond cerebral cortex including reticular thalamic nucleus, basal ganglia, and cerebellum, as well as in the peripheral nervous system (18). Perhaps a more efficient guide RNA/Cas9 combination would prove more effective in the *Scn8a*^W/+^-EIIa mice.

In the less severely affected *Scn8a*^W/+^-EMX1 mice, the base editor improved median survival from approximately 3 weeks, as observed in sham-treated mice, to over 3 months. Base editor-treated *Scn8a*^W/+^-EMX1 mice also exhibited fewer seizures than sham-treated counterparts, and the electrophysiological properties of neocortical excitatory glutamatergic neurons were normalized (16). Although seizures are the most prominent feature of *SCN8A*-DEE, the nonseizure comorbidities are a significant burden on patients and their families. These comorbidities include hypotonia, movement disorder, and intellectual disability/developmental delay (19). Few behavioral abnormalities have been identified in the *Scn8a*^W/+^-EMX1 mice, although in the open field test, Reever et al. observed a partial restoration of distance traveled across (a measure of hyperactivity) and time spent in the center (an index for anxiety).

## On deck: unanswered questions and future directions

A major safety concern of base editing is the potential for editing at off-target sites and “bystander” editing at like nucleotides adjacent to the target base. The gold-standard approach for off-target screening is to identify the most likely off-target sites using an unbiased method (e.g., CIRCLE-seq (20)) and subject these loci to deep sequencing. Reever et al. instead performed RNA-seq and whole-genome–seq (16). This method has the advantage of identifying potential off-target editing at any genomic location, but it is less sensitive than targeted sequencing of previously identified risk loci. The authors reported no significant off-target or bystander editing, but deeper sequencing of selected sites could have more rigorously identified potentially problematic events.

Reever et al.’s work represents what is, to our knowledge, the first published attempt to treat a genetic epilepsy using base editing (16). This is an exciting step toward a disease-modifying therapy for a severe condition that is typically refractory to conventional antiepileptic drugs and is associated with significant morbidity and mortality. However, many questions remain to be answered. Why was treatment of the global mutant not effective, and how can this be improved? What proportion of cells need to be corrected to improve epilepsy, nonepilepsy comorbid conditions, and survival, both in a global mouse model and in humans? What specific cell type(s) and brain regions need to be corrected for meaningful efficacy? Targeting the entire human brain with high efficiency will be difficult, so identifying discrete high-yield brain regions and/or particular cell types for correction could be both effective and practical.

While we still await a home run for base editing in neurological diseases, Reever et al. have put a runner on first base for the epilepsy field.

## Funding support

This work is the result of NIH funding, in whole or in part, and is subject to the NIH Public Access Policy. Through acceptance of this federal funding, the NIH has been given a right to make the work publicly available in PubMed Central.

- Brody Family Medical Trust Fund Fellowship from the Philadelphia Foundation to SFH.
- NINDS R01NS119977 and R01NS110869.
- A research grant from the Dravet Syndrome Foundation to EMG.

## Version 1 02/02/2026

Electronic publication
